# Founders’ Creativity, Business Model Innovation, and Business Growth

**DOI:** 10.3389/fpsyg.2022.892716

**Published:** 2022-06-10

**Authors:** Yang Li, Beiwei Li, Tianhao Lu

**Affiliations:** School of Management, Jilin University, Changchun, China

**Keywords:** creativity, business model innovation, business growth, empirical research, work experience

## Abstract

Given the existing studies on the role of different founders’ personalities in the growth of new ventures, we take the digital technology start-ups as the research object and focus on the role of founders’ creativity. In this study, we assess the relationship between founders’ creativity and business growth. According to the framework of upper echelons theory, we propose several hypotheses. Based on the investigation of 153 new ventures in China’s transition economy, we find that: (1) founders’ creativity has a positive impact on the growth of enterprises; (2) business model innovation positively mediates the relationship between founders’ creativity and enterprise growth; (3) work experience is found to be a moderator of the relationship between founders’ creativity and business model innovation. The conclusion of our analysis not only helps to further deepen the research on the growth process of start-ups but also helps to further expand the important role of business model innovation and founders’ work experience in the growth of new firms.

## Introduction

Business model innovation has received a lot of attention from scholars and is considered to be a key driver of new venture growth and sustainable competitive advantage. The study points out that the business model of enterprises is not static, especially in today’s international context of technological progress and rapid changes in market demand (Onetti et al., 2012; Wu, 2021). In entrepreneurial practice, more and more enterprises rely on business model innovation. In entrepreneurial practice, more and more enterprises rely on business model innovation to obtain corporate performance. For example, the domestic retail giant Suning Corporation, driven by Internet technology, has broken through the business model of traditional retail services, integrated into the e-commerce thinking, and gradually defined itself as “Suning Cloud Business,” leaving Gome Electrical Appliances in the same industry behind.

Some studies have pointed out that business model innovation is even more valuable than technological innovation to some extent (Teece, 2010; Bhatti and Danilovic, 2018), because business models are an important bridge for transforming new technologies into economic value, and through business model innovation, they promote the efficient commercialization of technology, thus bringing about the rapid growth of enterprises (Chesbrough, 2007). Especially in a dynamic environment, business model innovation helps companies cope with complex changes in external markets (Smajlović et al., 2019). It is precisely because of the important value of business model innovation that more scholars focus on the factors that promote/hinder business model innovation (Chesbrough, 2010; Bhatti and Danilovic, 2018; Snihur and Zott, 2020), such as the original value allocation mode of enterprises, technical conditions, leadership characteristics, and organizational changes, organizational learning (Sosna et al., 2010; Koch et al., 2018), changes in the market environment, value networks (Smajlović et al., 2019). Previous studies have mainly analyzed the role of organizational factors or external environmental factors on business model innovation. In recent years, scholars have gradually discovered that the founders of enterprises play a key role in the business model design process (Smith et al., 2010; Svejenova et al., 2010). Although scholars have explored the role of entrepreneurs’ entrepreneurship, background characteristics, social capital, and behavior in business model design (Bhatti et al., 2021; Zhao et al., 2021), the mechanism of how entrepreneurs’ characteristics influence business model innovation and lead to firm growth has been neglected.

To address this theoretical gap, this study focuses on the core characteristic of founders’ creativity. Existing research has found that the creativity of individuals (founders) contributes to the formation of entrepreneurial intentions (Hu et al., 2018), opportunity identification, and enterprise development (Dayan et al., 2013; Shane and Nicolaou, 2015; Farsi et al., 2019), business survival/growth (Mambula and Sawyer, 2004; Zhang et al., 2018), new product development (Im and Workman, 2004; Castillo-Vergara and Garcia-Perez-de-Lema, 2021). There is a lack of research on the impact of creativity on firm growth from the perspective of business model innovation. According to the Upper Echelons theory, top managers will give personalized explanations of the situation they face, thus making strategic decisions that lead to the growth of the enterprise (Hambrick and Mason, 1984). Therefore, this study has important theoretical value and attempts to solve the following two key problems through large-scale questionnaire surveys and empirical analysis: (1) how founders’ creativity and business model innovation affect enterprise growth, (2) the mediating role of business model innovation in the process of founders’ creativity affecting enterprise growth.

As mentioned above, founder creativity provides the premise for business model innovation and makes the team have more basis and willingness for innovation. However, the founder’s experience is also crucial to innovation decisions. Work experience usually refers to the experience that entrepreneurs have accumulated in their previous work fields, including knowledge of finance, marketing, management, etc. (Shane, 2001). The high level of work experience reflects that founders are better at seizing market opportunities and taking advantage of market conditions, and can identify more valuable opportunities for innovation and increase the possibility of innovative business models. Therefore, this study further explores the moderating effect of founders’ work experience on the relationship between their creativity and business model innovation.

In summary, we establish a novel framework for studying the impact of creativity on firm growth, with special attention to the mediating role of business model innovation and the moderating role of work experience. This study aims to make several contributions to the existing literature. First, this study contributes to the current literature by studying business model innovation as an intermediate process between SME creativity and firm growth. It also responds to Baron and Tang’s (2011) call to explore the mechanisms by which individual entrepreneurs encourage innovation at the firm level. Secondly, this study empirically examines the potential relationship between creativity and firm growth, thereby contributing to the literature on business model innovation by identifying the antecedents of business model innovation. Third, given the lack of research on boundary conditions of the relationship between entrepreneurial creativity and business model innovation, this study adds to the research on business model innovation by examining work experience as a moderator, which may promote or limit the impact of creativity on business model innovation.

## Theoretical Background and Hypotheses

### Founders’ Creativity

Creativity is considered by scholars to be a common trait possessed by successful founders (Pretorius et al., 2005) that helps inspire founders to generate new ideas and turn the idea into business opportunities to be exploited (Gielnik et al., 2012). Creativity is a complex and important business asset (De Miranda et al., 2009), helping founders cope with dilemmas such as a fiercely competitive environment, a changing market, and new technology needs. Creativity drives innovative activities in business and is considered a significant source of innovative activities to promote the development of new products/services, and organizational innovation (Cook, 1998; Heye, 2006).

As an individual-level concept, Perry-Smith (2006) defines creativity as an individual’s ability to generate novel ideas, new products, and solutions. Munizu and Hamid (2018) studied founders as the object of study, believing that creativity is a key skill possessed by founders. Creativity is an important source of innovation that drives the development of new businesses and new products/services. Research by Ahlin et al. (2014) argues that creativity is a personal trait that founders have. It shows that founders can often generate novel ideas and are good at using innovative ways to solve problems.

Founder creativity refers to personal traits that founders have. It shows that founders can often generate novel ideas and are good at using innovative ways to solve problems (Ahlin et al., 2014). It is an innate trait of the founder. The founder’s cognitive style is an individual difference in the preferred way of organizing and processing information and experience (Messick, 1996), which is an acquired ability and influenced by work experience and education level.

This study summarizes the views of relevant scholars and defines creativity as an important characteristic (ability) of founders to generate novel ideas and inventively solve problems.

### Business Model Innovation

The concept of business model innovation stems from scholars’ exploration of business models. The business model is to design the transaction content, transaction structure, and transaction governance of the enterprise to realize value creation by taking advantage of business opportunities (Zott et al., 2011). Business model innovation is seen as a systematic solution to overcome obstacles. However, it is understudied, especially in emerging economies (Xu et al., 2021). Business model innovation is the redesign of existing business models, involving the establishment of new value propositions, value creation systems, and value acquisition mechanisms to achieve business model re-engineering (Teece, 2010; Schneider and Spieth, 2013).

In terms of conceptual definition, Schneider and Spieth (2013) believe that business model innovation is the purpose of changing the existing strategic planning of enterprises, transforming and reconstructing the business management model, and achieving the purpose of enhancing corporate value and promoting enterprise growth. Aspara et al. (2011) believe that business model innovation is the process innovation of enterprises for potential customers, including business process and channel innovation.

The business model as the basic logic and strategic choice of the enterprise (Shafer et al., 2005), mainly revolves around value capture and value creation. It is widely accepted that business model innovation is regarded as an innovation in value creation, value proposition, and value acquisition (Guo et al., 2015; Clauss, 2017; Yang et al., 2017). Value creation innovation refers to the innovation of resources and capabilities used by enterprises to create value in the value chain (Achtenhagen et al., 2013), including new capabilities, new technologies or equipment, new partners, and new processes. Value proposition innovation is the innovation of solutions provided to customers and the way of presenting solutions (Morris et al., 2005; Johnson et al., 2008), including new products, new channels, and new customer relationships. Value acquisition innovation refers to innovation that translates value propositions into revenue (Baden-Fuller and Haefliger, 2013), including a new profit model, and a new cost structure (Clauss, 2017).

### Business Growth

The growth of new ventures has received increasing attention from scholars (Lee and Tsang, 2001; Gilbert et al., 2006). Because the environment in which new companies operate is rapidly changing, focusing solely on corporate performance does not objectively reflect the true state of the enterprise, while the growth of the enterprise can reflect the success of the enterprise (Lee and Tsang, 2001; McGrath, 2010). New ventures refer to enterprises that have not been established for a long time and have not yet gotten out of the survival dilemma, nor have they reached a stage of standardized and professional management. Enterprises in this stage are characterized by small scale, inexperience, and strong flexibility. Innovative ideas are the basis for the survival of these enterprises, which can play an effective role once they create an exploratory business model suitable for the internal development of the enterprise. At the same time, as a new venture is managed by an entrepreneur, the values of the enterprise are also concentrated on the entrepreneur. Therefore, we choose such enterprises as the target of our research to highlight the role of founders and business model innovation in the growth of enterprises. Business growth measures the state of the company in terms of sales revenue, employee growth, and market share (Gilbert et al., 2006). Creativity, as an important business asset for business success (De Miranda et al., 2009), influences the growth of business.

**FIGURE 1:**
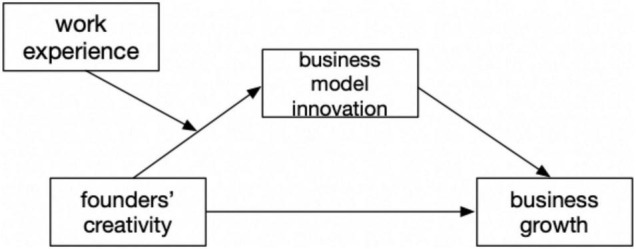
Conceptual framework.

Studies have shown that managers’ creativity has a significant impact on business growth (Bilton, 2010), Smith et al. (2010) have found that creativity has an important impact on the creation and growth of the business. However, his research mostly has focused on the study of team creativity and organizational creativity. Founders are an important driver of employee creativity and their relationship to enterprise growth has not been extensively studied. In recent years, business model innovation has become an important way for companies to achieve rapid growth (Zott and Amit, 2013). It has been a huge success for businesses by influencing their marketing models, and changing stakeholder relationships and corporate strategy (Cortimiglia et al., 2016). The innovative behavior of enterprises depends on the creativity of founders, business model innovation as a form of innovation, requires the role of creativity, based on this paper proposes the following theoretical model, see Figure 1.

### Founder Creativity and Business Growth

Studies have shown that the growth of a business is closely related to the creativity of its founders. Creativity is reflected in the process of founders creating and growing new businesses, and they are good at using novel ideas or ideas to gain an advantage for the business. Research by Bilton (2010) argues that the presence of creativity helps individuals to actively seek out new business opportunities and make more valuable discoveries that drive the creation of new value and maintain competitive advantage. Therefore, the creativity of this founder is an important driving force for the long-term development of the enterprise.

In the context of China’s emerging economy, the growth of enterprises is facing more uncertainties. The research by Cai et al. (2020) shows that opportunity development in this context requires the founder to have a certain degree of creativity. By using novel thinking and innovative ways, founders can solve the problems faced by enterprises to achieve growth. Including fierce market competition, shortage of resources, and lack of experience. Therefore, rich creativity helps to generate new ideas and propose creative solutions to problems. Through the improvement of product quality and the improvement of the marketing model, firms will win the recognition of customers and thus increase market share (Kilgour and Koslow, 2009).

Empirical research by Im and Workman (2004) confirms that creativity is positively correlated with the growth of a company’s sales revenue. The creativity of the founders also contributes to the formation of an innovative culture and atmosphere within the enterprise. The stronger an individual’s creativity, the greater the creativity of his team. Geroski et al. (2009) surveyed 529 manufacturing companies and found that companies with a creative culture are more profitable and grow faster. Because creative culture enables companies to innovate their products, services, and technical processes. They can use new methods to improve operational efficiency and reduce operating costs based on meeting the changing needs of customers (Aldrich and Martinez, 2007). Based on the above analysis, this paper proposes the following assumptions.

*H1: Founders’ creativity is positively related to business growth.*

### Business Model Innovation and Business Growth

Many studies have focused on the impact of business model innovation on enterprises. It is described as a highly innovative and exploratory process (Johnson et al., 2008). Unlike technological innovation or product innovation, business model innovation is essentially the uninterrupted discovery and development of opportunities (Grewal and Tansuhaj, 2001), which influence business growth in many ways.

Pohle and Chapman (2006) study IBM and discover that business model innovation can bring many benefits to the business including reducing the cost of the enterprise and rationalizing the cost structure of the enterprise. At the same time, this kind of innovative activity reduces the risk of investment and brings new market opportunities to companies. Amit and Zott (2001) believe that business model innovation is the source of value creation for companies and their suppliers, partners, and customers. Especially when the external environment is highly uncertain and difficult to predict. On the one hand, business model innovation activities help enterprises to resist potential risks. On the other hand, enterprises through value creation innovation, value proposition innovation, and value acquisition innovation take advantage of new opportunities to fully tap the resources and capabilities of enterprises to help enterprises gain a competitive advantage (Casadesus-Masanell and Zhu, 2013) to promote business growth.

Sorescu et al. (2011) studied business model innovation in retail and found that the value proposition outlines how companies create value through activities related to product development and pricing. Value capture outlines how companies can be governed, and reduce opportunity costs. By illustrating the way how value creation and value capture are created, the sources of competitive advantage can be more clearly described. By focusing on the value of the customer, managers are constantly adapting to the changing needs of customers from an outside-in perspective (Sorescu et al., 2011). The growth of enterprises can be realized through innovative value propositions. Therefore, through innovative value proposition, value capture, and value creation, we can implement product iterations, and cost savings and gain competitive advantages for enterprises to drive enterprise growth.

Business model innovation enables firms to establish network boundaries that allow them to explore opportunities and capture value more effectively (Zott and Amit, 2007, 2013). Research by Pries and Guild (2011) confirms that business model innovation helps companies turn new products or technologies into revenue on time. Latifi et al. (2021) studies have found that BMI can affect the efficiency, organizational ability, and income of enterprises, thus affecting the growth of enterprises. Chen et al. (2021) find out growth is also achieved through the indirect effect of business model innovation on customer trust and commitment. Therefore, business model innovation can maintain the sustainable growth of enterprises through multiple paths. Paths include seizing opportunities in volatile environments, reducing costs, and increasing flexibility (Pohle and Chapman, 2006; Bock et al., 2012), and leveraging existing systems of resources and capabilities (Schneider and Spieth, 2013). Based on this, this study proposes the hypothesis:

*H2: Business model innovation is positively impacting business growth.*

### Founders’ Creativity and Business Model Innovation

Creativity is a prerequisite for innovation (Anderson et al., 2014). Innovation is defined as the generation of creative ideas and the implementation of these ideas into “new products, new services, new processes” (Amabile, 1988; Amabile et al., 1996). Business model innovation takes advantage of new opportunities and represents a comprehensive change in the organization (Chesbrough, 2010; Demil and Lecocq, 2010; Guo et al., 2015; Zhang et al., 2018).

Creative leaders will give more encouragement and support to the team, and the team led by them has more behavioral factors that encourage creativity (Amabile et al., 1996). At this point, creative behavior manifests itself among managers and their teams, creating interactions that translate ideas into new products, services, processes, markets, and even suppliers (Johannessen et al., 2001). Creative teams generate more ideas, analyze the most promising ideas and implement product and service innovations, thus creating value for the organization (Gundry et al., 2016).

Founders directly determine the authorization and creation of enterprises, so their decision-making power plays an important role in the business model innovation of enterprises (Kaner, 2014). Founders’ creativity leads to innovative business models through exploration. March’s (1991) study found that incremental improvements to existing ideas and breakthroughs from new ones were necessary to sustain and renew the business. This helps organizations maintain deliverability and develop new or even conflicting business models while maintaining existing ones (Johnson et al., 2008).

*H3: Founders’ creativity is positively related to business model innovation.*

### Mediating Role of Business Model Innovation Between Founders’ Creativity and Business Growth

Creativity represents the typical individual characteristics of founders who indirectly influence corporate output through a series of actions or processes (Frese, 2009), where innovation is the critical path. For example, Hon and Leung (2011) argue that creative founders apply new ideas and create an atmosphere and culture of innovation within the organization. The culture of innovation is a key influential factor in achieving resource optimization and organizational process change. A culture of innovation drives changes. Innovative organizational culture helps members of the organization to participate in the development of new products, and provide new products and services to customers (Claver et al., 1998; Perry-Smith, 2006).

Creativity also helps to break down the cognitive barriers of founders. The founder’s motivation to innovate in the business model is suppressed by the forces of inertia because the founder has cognitive impairment (Chesbrough, 2010) and the founder has a continuity of behavior patterns with his employees. As a result, existing business models discourage founders from changing the logic of existing value creation and value acquisition (Huang et al., 2013). Mayfield and Mayfield (2008) argue that entrepreneurial creativity can break inertia by eliminating oneself and employees’ fears of change and innovation, and by making employees more positive about innovative behavior. Garfield et al. (2001) propose a creativity support system within the organization, which is used as a tool for enterprise innovation to facilitate the formation of innovation by stimulating individuals and eliminating individual cognitive inertia. Therefore, creativity can break the inertia of the original business model to promote innovation, thereby promoting business growth.

Individual-based creativity can induce founders to generate new ways of value creation and value propositions. On the one hand, rich creativity helps individuals to think about how to efficiently use the resources and capabilities of the enterprise. Through creative resource integration, the originally inactive resource elements and material assets are activated and transformed into corresponding values. On the other hand, creativity helps to discover the diverse needs of customers (Horn and Salvendy, 2006). Creative individuals place more emphasis on investing in human and social capital (Makri and Scandura, 2010). Individual creativity positively influences firms and helps them build stable customer relationships, supplier networks, and other partnerships by making stakeholders feel the novelty and adaptation of the work or service (Lepak et al., 2007). Therefore, unleashing the creativity of founders not only fully captures customer needs, but also helps companies discover new ways and new ways of creating value for stakeholders (Casadesus-Masanell and Zhu, 2013). Help companies form new value propositions by discovering competitors’ market positioning, products, and services, strengths and weaknesses. That is, the formation of new business models (Shu et al., 2012; Clauss, 2017). These innovative business models drive companies to better identify and capitalize on opportunities (Grewal and Tansuhaj, 2001; Xiao et al., 2021), meeting customer and market needs to bring about rapid growth (Zott and Amit, 2010). Creativity also contributes to the formation of novel and meaningful ideas that are necessary for innovation (Amabile, 1988). That is, creativity is conducive to enterprises producing novel ways of value creation, thereby improving corporate performance and bringing about corporate growth. Based on the above analysis, this study proposes the hypothesis:

*H4: Business model innovation mediates the relationship between founders’ creativity and business growth.*

### Moderating Role of Work Experience

Work experience is the accumulated experience of the entrepreneur in the previous work field (Shane, 2001). In the study of work experience, researchers take the working experience and working years of entrepreneurs as important standards to measure work experience. Previous studies have found that there is a positive correlation between work experience and individual performance. Work experience is closely related to the formation of entrepreneurs’ implicit knowledge (Wu and Ma, 2018), which affects the achievement of individual core work tasks.

The influence of work experience on innovation and entrepreneurship activities has been concerned by many scholars. According to the research, entrepreneurs’ previous work experience includes knowledge of finance, marketing, management, etc., which can significantly enhance entrepreneurs’ identification of opportunities (Shane, 2001). In other words, entrepreneurs’ work experience has accumulated knowledge for entrepreneurs to carry out innovative activities, and this knowledge will affect entrepreneurs’ identification of subsequent opportunities. A high level of work experience will bring a high level of opportunity identification ability, which can help enterprises understand and grasp the market trend, grasp the potential market demand, and take targeted activities to respond to the market environment. Especially in the context of a transition economy, various available business opportunities provide important opportunities for business model innovation. Under the tide of the digital economy, it has brought great influence to the reform of the business model.

In addition, Shan and Lu (2020) found that entrepreneurs’ entrepreneurial experience has a positive impact on experiential learning, which further supports the importance of experience in entrepreneurial activities. However, entrepreneurial experience does not always lead to positive results for new businesses. The key to using it effectively is to learn from experience and turn it into entrepreneurial knowledge. Entrepreneurial experience and managerial experience enhance the complexity of their business planning (Ma et al., 2021).

As a personality trait of entrepreneurs, a founder’s creativity will influence the formation of innovative ideas, and thus affect their innovative decisions for enterprises. Work experience provides entrepreneurs with commercially valuable information and knowledge about markets, and products, and enables entrepreneurs to effectively synthesize situations to identify innovative ideas. It plays a complementary role in their innovation decisions, which may strengthen the relationship between creativity and business model innovation.

*H5: Work experience moderates the relationship between founders’ creativity and business model innovation. Compared with a low level of work experience, the positive correlation between founders’ creativity and business model innovation is stronger under the influence of a high level of work experience.*

### Moderated Mediating Effect

Hypothesis 4 explains the mediating effect between founders’ creativity and business growth, and hypothesis 5 explains the moderating effect of work experience on the relationship between founders’ creativity and business model innovation. Based on the above discussion, this paper proposes a moderated mediation model to explore the influence mechanism and conditions of founders’ creativity on business model innovation. When founders have higher work experience, the positive correlation between creativity and business model innovation is stronger, and the impact of creativity transmitted through business model innovation on enterprise growth is correspondingly stronger. Therefore, the following hypothesis is proposed.

*H6: Work experience positively moderates the indirect effect of founders’ creativity on business growth through business model innovation. That is, the higher the Work experience, the greater the mediating effect of business model innovation.*

## Materials and Methods

### Sample and Procedure

The study tested the theoretical model using data from Chinese start-ups in the Yangtze River Delta region, including Beijing, Tianjin, Shanghai, and Suzhou. Participants are recruited as follows. First of all, MBA students from Jilin University and Zhengzhou University are sent questionnaires by email to their companies. At the beginning of the questionnaire, we explain the research procedures and emphasized that the research is conducted for academic purposes under the condition of complete confidentiality. We connect founders and other managers through their executives. The questionnaire is filled out by the founder and then by other senior executives of the company, such as the CMO or the CEO.

Two sets of questionnaires are designed for this study. The questionnaire research consists of two stages: in stage 1, the founders of enterprises need to complete the questionnaire survey on the predictive variables (individual creativity level) and control variables (age, gender, and education level) at the individual level. One month later, in stage 2, other executives were asked to complete enterprise-level questionnaires on dependent variables (enterprise growth), mediating variables (business model innovation), and team-level control variables (enterprise size, enterprise establishment time).

Finally, A total of 192 enterprises were surveyed, including 405 participants. Four hundred and five individual-level questionnaires and 192 team-level questionnaires are collected. Among them, 145 questionnaires at the individual level and 39 questionnaires at the team level were discarded due to lack of data, leaving 153 sets of valid questionnaires at the individual level and team level. Among them, nine questionnaires were removed because of their establishment years. Thirty were submitted because their answers were incomplete.

The sample characteristics are as follows: 73.86% for technology enterprises, and 26.14% for no-technology enterprises. In terms of ownership, 66.01% of the samples were private or holding enterprises. In terms of enterprise size, 45.10% of the sample of enterprises with less than or equal to 50 employees, 54.90% of the sample of enterprises with more than 50 employees. The average age of companies was 8.82 years. The characteristics of samples are shown in Table 1.

**TABLE 1:** Characteristics of samples.

Characteristics	*N*	Percentage (%)
**Industry type**		
Technology-based industry new ventures	113	73.86
Other industry new ventures	40	26.14
**Company age**		
1–8 years (including 8 years)	85	55.56
Over 8 years	68	44.44
**Firm size (employees)**		
01–20	46	30.07
21–50	23	15.03
51–200	53	34.64
Over 200	31	20.26

### Measures

All scale items are originally developed in English and therefore translated into Chinese. Likert 7-point scale is used for all scale items, ranging from 1 = “strongly disagree/unlikely” to 7 = “strongly agree/probably.”

### Enterprise Growth

The scale of enterprise growth is adopted from Gilbert et al. (2006). The three types of questions are measured as follows: (1) The growth rate of enterprise sales revenue. the growth rate of enterprise market share. (2) The growth rate of the number of employees in an enterprise. (3) The interviewees will evaluate according to the actual situation of the enterprise. The Cronbach’s alpha for the scale of enterprise growth is 0.733.

### Founders’ Creativity

Founder creativity is measured with four questions and developed by Khedhaouria et al. (2015). The items such as (1) founders have the confidence to solve problems creatively. (2) Founders are good at developing original ideas. (3) Founders tend to try new approaches at work. (4) Founders play a creative role well. The Cronbach’s alpha for this scale is 0.891.

### Business Model Innovation

Business model innovation includes three categories: value creation innovation, value proposition innovation, and value capture innovation. Business model innovation is measured with three topics scale developed by Clauss (2017). The nine items for measuring value create innovations as follows: *New Capabilities.* (1) Our employees are constantly trained to develop new capabilities. (2) We constantly reflect and think about the latest capabilities needed to cope with market changes. *New Technologies (New Equipment)* (3) Our company’s technical resources are up to date. (4) We often take advantage of new technology opportunities to expand our portfolio of products and services. *New Partner.* (5) We are always looking for new partners. (6) We often take advantage of opportunities arising from the integration of new partners into us. (7) New partners often help us develop our business model further. *New Process.* (8) We use innovative procedures and processes in the manufacturing of our products. (9) We regularly review existing processes and make major changes as needed. The Cronbach’s alpha for the scale of value creates innovations is 0.936.

The seven items for measuring value proposition Innovation are as follows: *New Supply.* (1) We often deal with new, unmet customer needs. (2) Our products and services are innovative, and we usually solve problems that our competitors can’t solve. *New Customers and Markets.* (3) We are often able to seize opportunities that arise in new or growing markets. (4) We constantly seek new customer segments and markets for our products and services. *New Channel.* (5) We often use new distribution channels for our products and services. *New Customer Relationships* (6) We try to increase customer retention by offering new services. (7) We emphasize innovation/modern actions to improve customer retention. The Cronbach’s alpha for the scale of value proposition Innovation is 0.917.

Value capture innovation is measured with four items. *New Profit Model*. (1) We have recently developed new revenue opportunities (e.g., additional sales, cross-selling). (2) We are increasingly offering integrated services (such as maintenance contracts) to achieve long-term financial returns. (3) We recently supplemented or replaced one transaction revenue with a long-term recurring revenue model such as leasing. *New Cost Structure.* (4) We actively look for opportunities to save manufacturing costs. The Cronbach’s alpha for this scale is 0.878.

### Control Variables

Previous studies have shown that demographic variables and enterprise characteristics may affect team creativity. Including the size of the company, age of the company, and entrepreneur’s education level. Because of the different sizes of the enterprise, the founders have different control over the enterprise. Similarly, the different establishment time and operation mechanism of enterprises affect the degree of influence of creativity on business model. Therefore, we set enterprise-scale and establishment time as control variables at the enterprise level. At the individual level, we take into account the education level of the founders, because the education level will affect the founders’ work attitude, values and management level (Hambrick and Mason, 1984). Therefore, these variables are controlled in this research. Enterprise-scale is divided into four levels according to the number of employees (1 = less than 20 employees, 2 = 20–50 employees, 3 = 50–200 people, 4 = more than 200 people). Company age refers to the number of years of registration at the time of the survey. Entrepreneur’s education is divided into four levels (1 = high school or below, 2 = junior college, 3 = bachelor degree, 4 = master degree or PhD).

### Common Method Bias

According to Podsakoff and Organ (1986), Harman’s single factor test is used to examine the problem of common method bias (CMB). Principal component factor analysis shows that no single factor can explain most of the variance. The largest factor only accounted for 23.51% of the variance. Therefore, CMB did not appear in this study.

### Data Analysis

To analyze the validity of the questionnaire, this study uses the statistical software SPSS 25.0 to analyze the obtained sample data: By KMO test and Bartlett sphericity test, the results show that KMO = 0.911 and Bartlett test is significant at *P* < 0.001 level.

The reliability and validity test results of core variables, founders’ creativity, business model innovation, work experience, and enterprise growth are shown in Table 2. It can be seen from Table 2 that the factor values of the questions corresponding to each variable are all above 0.7 (only one-factor value is 0.62). Cronbach’s alpha coefficients involved in the reliability test are all more than 0.8. The combined reliability CR is above 0.7 and AVE is more than 0.5, indicating that the scale had good internal consistency and convergence validity. This fully shows that the questionnaire has good validity and meets the requirements of further data analysis.

**TABLE 2:** Reliability and validity test results.

Variable	Index	Estimate	Cronbach’s *a*	CR	AVE
Business growth	NG1	0.878	0.733	0.856	0.670
	NG2	0.620			
	NG3	0.924			
Founders’ creativity	FC1	0.910	0.891	0.927	0.761
	FC2	0.864			
	FC3	0.807			
	FC4	0.904			
Business model innovation	BMI1	0.774	0.936	0.955	0.603
	BMI2	0.837			
	BMI3	0.841			
	BMI4	0.817			
	BMI5	0.803			
	BMI6	0.825			
	BMI7	0.853			
	BMI8	0.779			
	BMI9	0.792			
	BMI10	0.764			
	BMI11	0.780			
	BMI12	0.860			
	BMI13	0.767			
	BMI14	0.852			
	BMI15	0.863			
	BMI16	0.853			
	BMI17	0.838			
	BMI18	0.837			
	BMI19	0.922			
	BMI20	0.828			

### Correlation Analysis and Hypothesis Testing

The results of descriptive statistics and correlation analysis (Pearson) are shown in Table 3. Describe descriptive statistics and correlations between the main variables used in the regression analysis. We examined the multicollinearity problem carefully. Pearson correlation coefficients between these core variables were all less than 0.6. Then the variance inflation factor (VIF) is calculated. All VIF results were below 10. Hair et al. (1998) showed that there was no significant multicollinearity.

**TABLE 3:** Descriptive statistics and correlation matrix for the variables.

Variables	M	SD	1	2	3	4	5	6	7
Work experience	9.480	6.196	1						
Entrepreneurial education	2.840	0.954	–0.172	1					
Employee number	2.930	1.015	0.356**	0.039	1				
Enterprise age	9.182	6.369	0.427**	–0.068	0.474***	1			
Founders’ creativity	4.763	1.146	0.237***	–0.124	0.036	0.199*	1		
Business model innovation	5.230	0.925	0.115	−0.231*	–0.037	0.055	0.526***	1	
Business growth	4.789	1.116	0.265**	−0.221*	0.139	0.112	0.350***	0.698***	1

***p < 0.001, **p < 0.01, *p < 0.05.

We use hierarchical linear analysis to test models and hypotheses. The results are shown in Table 4. Model 1 shows the influence of control variables on enterprise growth. The results show that except for education level, other control variables do not influence enterprise growth. Model 2 is established to verify the main effect. The results showed that the coefficient of creativity is 0.358 (model 2: β = 0.358; *p* < 0.001). The results show that creativity has a positive impact on enterprise growth. Therefore, hypothesis 1 is supported by the sample.

**TABLE 4:** The results of regression analysis.

	Business growth				Business model innovation	
Variables	Model 1	Model 2	Model 3	Model 4	Model 5	Model 6
**Control variables**						
Age	–0.004	0.032	0.146**	0.137*	–0.133	–0.038
Employee number	0.035	0.002	–0.029	–0.026	0.035	0.003
Entrepreneurial education	0.240**	0.16	0.099	0.102*	0.072	0.104*
**Independent variables**						
Founders’ creativity		0.358***		–0.070	0.540***	0.624***
**Mediating variables**						
Business model innovation			0.757***	0.792***		
**Moderating variables**						
Work experience						0.028
Work experience × FC						0.211***
R2	0.119	0.233	0.644	0.649	0.337	0.383
Adj. R2	0.081	0.191	0.625	0.626	0.301	0.324
F value	3.138*	5.587***	33.703***	28.072***	9.349***	9.526***

***p < 0.001, **p < 0.01, *p < 0.05.

Model 3 verifies the impact of business model innovation on enterprise growth. The results show that the coefficient of business model innovation is 0.757 (model 3: β = 0.757; *p* < 0.001), which verifies the positive effect of business model innovation on enterprise growth. Based on the results, we can confirm hypothesis 2. The results of model 5 show that founders’ creativity has a positive impact on business model innovation (model 5: β = 0.540; *p* < 0.001). Hypothesis 3 is confirmed.

We use the study of Baron and Kenny (1986) to examine the mediating role of business model innovation. We compared model 2 with model 4. The results showed that the regression coefficient of model 4 is –0.070 (model 4: β = –0.070; ns) are lower than that in model 2 (model 2: β = 0.358; *p* < 0.001). The results in Model 5 show that founders’ creativity has a positive impact on business model innovation (model 5: β = 0.540; *p* < 0.001). Therefore, business model innovation has a positively mediating effect between founders’ creativity and business growth. Based on the above analysis, hypothesis 4 is also supported by data.

**FIGURE 2:**
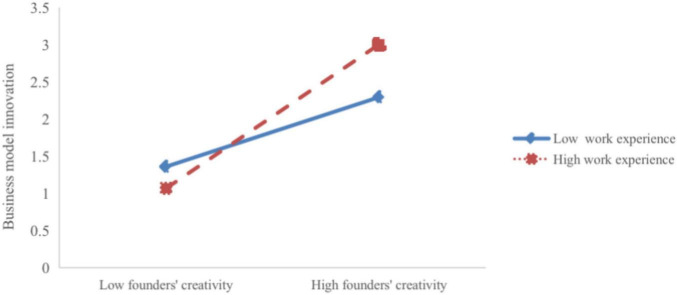
Interactive effect of founders creativity and work experience on business model innovation.

Testing the moderating effect of work experience. From model 6, it can be seen that the interaction term between the founder’s creativity and work experience (model 6: β = 0.211; *p* < 0.001) had a significant positive impact on business model innovation, suggesting that founders’ work experience enhanced the positive correlation between founders’ creativity and business model innovation. Figure 2 shows that when the founder has a high level of work experience, the founder’s creativity has a stronger correlation with business model innovation, and the slope is larger. Hypothesis 5 is supported.

In this paper, the Process program is used to test the moderated mediating effect and calculate the mediating effect under different levels of work experience. The results are shown in Table 5. It can be seen that there is a significant difference between the two indirect effects, indicating that the mediating effect of business model innovation is moderated by work experience under high and low working experience, and hypothesis H6 has been verified. In addition, according to the index, work experience moderates the mediating effect of business model innovation is 0.167, and the confidence interval does not include 0, which further indicates that work experience strengthens the mediating effect of business model innovation on founders’ creativity and enterprise growth.

**TABLE 5:** Results of moderated mediating effect test.

Mediator	Work experience	Effects	SE	95% confidence interval	Index	SE	95% confidence interval
BMI	M–1SD	0.295	0.086	[0.156, 0.496]			
BMI	M–1SD	0.455	0.077	[0.332, 0.631]	0.167	0.073	[0.036, 0.319]
BMI	M+1SD	0.615	0.120	[0.420, 0.884]			

## Discussion

This paper takes entrepreneurial enterprises as the research object and based on insufficient research on creativity and business model innovation, deeply discusses how creativity can influence business model innovation and bring enterprise growth. From the perspective of business model innovation, it reveals the key role path of the founder’s creativity in enterprise growth. The results of empirical analysis based on the survey samples in China show that a founder’s creativity has a positive impact on firm growth. Business model innovation plays a positive mediating role between founder creativity and firm growth. Work experience strengthened the positive correlation between creativity and business model innovation. Work experience reinforces the mediating role of business model innovation between creativity and firm growth.

The relevant conclusions make up for the deficiency of existing research. The empirical research results support the research model proposed in this paper, and the main findings are as follows:

First, we found that founders’ creativity is an important driving force for the growth of enterprises. Founder creativity is the unique ability to conceive novel ideas and identify valuable business opportunities (Heinonen et al., 2011; Tu and Yang, 2013; Siemon and Robra-Bissantz, 2018). Unlike the founder’s management skills, which keep a business on track, creativity is about thinking new and doing something new that no one else has done before. Creativity is more important than a rigorous attitude, disciplined management, corporate reputation, and even a vision of the future. It can guide enterprises to find the direction of success in an increasingly complex world, especially in unexpected changes (Sijabat et al., 2021). In the era of rapid change in enterprise products, homogenized competition, and limited resources force enterprises to use new thinking to win long-term development. The empirical research in this paper also well confirms this point of view.

Second, we found that business model innovation is the cornerstone of enterprise growth. Business model innovation is comprehensively reflected in putting forward new value propositions for organizations, designing novel value creation systems or improving original value capture mechanisms, and creating new or reshaping existing business models (Guo et al., 2015). These innovative activities will create new profit models and unique competitive advantages for enterprises, and lay a foundation for enterprise growth. Some enterprises through business model innovation, such as providing customers with novel products or services (Visnjic et al., 2016); new customer relationships (Baden-Fuller and Haefliger, 2013), developing new markets, establishing the organization to establish a unique ability to distinguish itself from other competitors. This is reflected in the organization to build a new profit model or optimize the cost structure, to create profits. Therefore, business model innovation promotes the long-term development of enterprises by establishing new logic and new ways of creating and acquiring value (Casadesus-Masanell and Zhu, 2013). All these provide references for enterprises in practice.

Third, business model innovation is an important way to give play to a founder’s creativity and realize enterprise growth. We further expand the feature-innovation-growth model and find that business model innovation is the mediation between the founder’s creativity and the firm’s growth (Laguir and Den Besten, 2016). Previous studies have shown that there may be a potential relationship between business model innovation and creativity. Taking a founder’s creativity as a part of entrepreneurship is an important antecedent of business model innovation because the decisions related to business model innovation are closely related to the founder’s creativity. Founders’ creativity helps to overcome the obstacles of thinking inertia (Mayfield and Mayfield, 2008), form an innovative organizational culture, discover business opportunities through creative thinking (Geroski et al., 2009) change organizational structure, explore new technologies and other ways, innovate business models to shape new competitive advantages and bring about enterprise growth.

Fourth, the indirect impact of founders’ creativity on firm growth is moderated by founders’ work experience. Based on the higher-order theory, low-level work experience may not be able to use their creativity, while high-level work experience may be better at using their creativity in business model innovation that is more conducive to the growth of the organization. In other words, founder work experience has a greater moderating effect on founder creativity and business model innovation. The greater the mediating effect of business model innovation on founder creativity and firm growth.

### Theoretical Application

An important theoretical contribution of this study is to reveal the role of founder creativity on business model innovation. Existing researches mainly discuss the connotation, process, and effect of business model innovation on competitive advantage, as well as the effect of organizational factors or external environmental factors on business model innovation. However, as the role of the founder, the core figure of new enterprises has been neglected, and some existing researchers have also emphasized that the relationship between creativity and enterprise innovation is not clear (Sarooghi et al., 2015). In particular, there is a lack of research on how its creative characteristics affect business model innovation. This study believes that founder creativity, as a special talent of founders, helps to promote founders to form a good organizational atmosphere for innovation in the process of entrepreneurship and realize business model innovation through organizational change and other ways. Relevant research conclusions make up for the deficiencies of existing theoretical research on business model innovation.

Another theoretical contribution of this paper is to explore the important mechanism of founder creativity on firm growth and reveal how founder creativity brings firm growth through business model innovation. Current research usually discusses the direct role of creativity and firm performance (Peljko et al., 2017), neglecting theoretical research on the path and mechanism through which creativity affects new firms. Based on the theoretical logic of “founder characteristics-behavior-firm output,” this paper systematically analyzes the internal relationship among founder creativity, business model innovation, and firm growth. This study proposes that creativity can bring sustainable competitive advantage and expand market share for enterprises by influencing the innovation of value proposition, value creation, and value acquisition, to realize enterprise growth. The research results help to make up for the lack of research on the role of founder characteristics on organizational behavior and firm growth.

Finally, work experience is incorporated into the theoretical analysis framework to reveal the boundary conditions of the impact of founder creativity on business model innovation, which is helpful to enrich the research on management autonomy. Current studies on the situational mechanism of the impact of creativity on business models pay little attention to the impact mechanism of work experience. From the perspective of management decisions, this paper explores the moderating effect of work experience on the relationship between creativity and business model innovation (Cooper and Bruno, 1977; Barringer et al., 2005). Research on the contingency mechanism of the influence of founder creativity, a personality trait, on business model innovation. Work experience provides managers with more information to make decisions, thus increasing the likelihood of successful innovation for founders.

### Practical Application

First of all, the research results show that creativity can affect the growth of enterprises in many ways. Therefore, it is wise for team founders to utilize their creativity and realize the growth of their companies. The creativity of founders helps enterprises to use innovative thinking to find opportunities that are hard to be seen by others, and promote the growth of enterprises by launching creative products or services that fully meet the needs of consumers. In addition, creativity can give entrepreneurial teams a good atmosphere for innovation and help enterprises update information and knowledge constantly according to the changes in the environment. In the practice of entrepreneurship, not all good managers are creative. Managing the enterprise well does not make the enterprise innovative, but innovation is an important factor driving the growth of the enterprise. Just as Silicon Valley has spawned countless excellent companies, their founders are more creative than they can manage, which is why Silicon Valley has become the most concentrated and successful place for entrepreneurship and innovation. Therefore, the founders of enterprises should pay attention to maintaining and cultivating their creativity, especially in the fierce dynamic competition environment, need to develop new ideas, find and create better opportunities, to continue to grow for the enterprise.

Second, enterprises should pay attention to model innovation under the new background. Innovation is not only reflected in technological innovation. Under the background of the “new normal” of China’s economy, traditional industries with homogenized product contents can give new vitality to traditional industries through business model innovation and integration with the Internet in the new situation. Taking books as an example, the value proposition it provides to customers is no longer just knowledge acquisition but brings new value appreciation to books by adding new propositions such as ornamental value and collection value. Traditional manufacturing enterprises, such as Nike, have achieved value co-creation through outsourcing. By outsourcing, Nike eliminates activities at the bottom of the value chain and streamlines the organizational structure to increase efficiency and reduce costs. When this value co-creation extends to the Internet era, that is, the business model of sharing platform is derived. When more and more new models are combined with traditional industries, a new round of growth advantages will be brought to traditional enterprises.

Third, as an important intermediary between founder creativity and enterprise growth, business model innovation is an effective tool for innovation of new enterprises in the new era. Chinese enterprises are facing the problem of enterprise transformation, but it is difficult to change the concept of entrepreneurs, especially the chairman and CEO of listed companies aged between 45 and 60, lack of motivation for transformation, “powerless” becomes an important obstacle to transformation. For founders who are focused on the long term, being creative is critical, because creativity is likely to be the engine of transformation. Some studies found through multiple case studies that when enterprises with rapid growth face model competition across multiple industries, founders with the motivation to disrupt the industry have the ability of discontinuous management, that is, creativity is the key to successful innovation of business model. The application of creativity to the innovation of business model does not require a large cost of reform, but it can give full play to the role of the founder’s new thinking and new decision, achieve breakthroughs in transaction mode, operation mode, and management mode, and reduce the risk of innovation investment. The innovation mode should be oriented by customer value, enhance the added value of products and seek opportunities from customer demand. This requires founders to use creativity to find and dig opportunities, innovate value propositions, update business objectives, change the way of acquiring value, and always develop new business models for the organization centering on customer value, thus bringing the growth of enterprises.

At the same time, Chinese enterprises have been at the bottom of the industrial chain for a long time. The innovation of the business model can help enterprises gradually occupy a favorable position and even lead the industrial format by extending the industrial chain. When the external environment is constantly changing, many enterprises are faced with the problem of transformation and development, which requires founders to carry out business model innovation with new ideas. In this process, founders should not only have the creative thinking to redefine customer value and create new rules, but also have the innovation ability to upgrade and reshape the business model, and gradually upgrade to the dual drive of “model innovation and technology innovation,” improve competitive advantages, to achieve long-term and stable growth.

Fourth, work experience moderates the influence of founder creativity and business model innovation. Team leaders should pay attention to the accumulation of work experience. On the one hand, it is the accumulation of industry knowledge. Having relevant industry experience will help entrepreneurs skillfully obtain industry development information, analyze industry changes, and better grasp market demand (Politis, 2005). On the other hand, it is the summary and learning of past work experience and entrepreneurial experience. These industry experiences can enhance entrepreneurs’ foresight and judgment on the development direction and uncertainty of the industry involved, thus reducing the materiality and uncertainty of entrepreneurs in the process of entrepreneurship.

### Limitations and Future Research

Despite the theoretical and practical contributions of this study, there are still some potential limitations. First, this paper only discusses the impact of the individual creativity of enterprise founders on the growth of enterprises, without discussing the role of other individual characteristics. Although this paper discusses the role of founders’ creativity in the growth of enterprises, founders’ decisions cannot completely control the direction of enterprises, and assertive behavior is not conducive to the development of enterprises. Other team members also play an important role in the development of the team. In the future, the interaction between founders’ other characteristics and creative characteristics as well as the interaction between founders and other members on the impact of corporate growth will be studied.

Second, the average age of the enterprises studied in this paper is 8.82 years old, and they are in different stages of enterprise development (growth, maturity, etc.). The application of founder creativity varies at different stages of an enterprise. The research conclusion of this paper provides a transformation path of business model innovation for enterprises that have established and want to obtain sustainable competitive advantages, but not all enterprises at all stages need the same degree of innovation. Future research will consider the different applications of founder characteristics at different stages of an enterprise.

Third, this research results indicate that in the Internet age, the vast majority of companies can grow through the innovative business model for enterprises, but the approach to innovation may be varied with the change in business environment and industry differences and different, therefore, future research should combine environment and industry background, has more practical meaning.

## Data Availability Statement

The raw data supporting the conclusions of this article will be made available by the authors, without undue reservation.

## Ethics Statement

The studies involving human participants were reviewed and approved by School of Management, Jilin University. The patients/participants provided their written informed consent to participate in this study. Written informed consent was obtained from the individual(s) for the publication of any potentially identifiable images or data included in this article.

## Author Contributions

YL developed theoretical models, wrote manuscripts, and was responsible for empirical analysis. BL collected and analyzed the data and participated in manuscript writing. TL co-wrote the manuscript. All authors contributed to the article and approved the submitted version.

## Conflict of Interest

The authors declare that the research was conducted in the absence of any commercial or financial relationships that could be construed as a potential conflict of interest.

## Publisher’s Note

All claims expressed in this article are solely those of the authors and do not necessarily represent those of their affiliated organizations, or those of the publisher, the editors and the reviewers. Any product that may be evaluated in this article, or claim that may be made by its manufacturer, is not guaranteed or endorsed by the publisher.
